# Communication and Emotional Vocabulary; Relevance for Mental Health Among School-Age Youths

**DOI:** 10.3389/fpsyg.2022.847412

**Published:** 2022-04-25

**Authors:** Tormod Rimehaug, Silja Berg Kårstad

**Affiliations:** ^1^Regional Centre for Child and Youth Mental Health and Child Welfare (RKBU Central Norway), Department of Mental Health, Faculty of Medicine and Health Sciences, NTNU – Norwegian University of Science and Technology, Trondheim, Norway; ^2^Department of Child and Adolescent Psychiatry, Nord-Trøndelag Hospital Trust, Levanger, Norway

**Keywords:** emotional vocabulary, communication, mental health, school-age, development

## Abstract

**Background:**

The association between language and mental health may be connected to several aspects of language. Based on the known associations, emotional vocabulary could be an important contribution to mental health and act as a risk, protective or resilience factor for mental health in general. As a preliminary test of this hypothesis, an assessment of emotional vocabulary was constructed and used among youths in school age. Cross-sectional associations and prediction models with parent-reported youth mental health as outcome were examined for emotional vocabulary as well as general vocabulary, non-verbal problem solving and social communication, controlled for age, gender and subsamples.

**Results:**

Emotional vocabulary, general vocabulary and non-verbal problem solving were directly associated with each other and similarly associated with age and gender. However, they were not significantly associated with social communication skills or mental health in the expected direction. Only social communication skills showed significant negative associations with behavioral mental health problems, suggesting these skills to be potential resources related to mental health.

**Implication:**

Future research should investigate whether behavioral problems may be prevented or improved by developing better social communication skills among community school-age youths. However, our results suggest that merely expanding emotional vocabulary is not likely to produce such effects unless this is integrated with improving social communication.

## Introduction

There is a widespread assumption that language is important for mental health in several ways; both by language influencing the development and improvement of mental health problems and social skills and social problems, but also by mental health influencing communication and communication skills (Pons et al., 2019). In these ways, typical development of language and communication is expected to be both a protective factor against mental health problems, and a resource for positive mental health like social functioning and cognitive problem solving. Nonetheless, language and communication can also increase the capacity for rumination and social misunderstanding and conflict, with negative consequences for mental health.

However, these assumptions have lacked systematic documentation, and disentangling which aspects of language and communication are most important is still in progress. The important aspects may be a rich and complex vocabulary, the understanding of emotional concepts and expressions, or the understanding and aiding of social processes and communication. This could point toward the idea that “more is better” or that all these aspects are equally important. In an intervention study with preschool/1.grade children a systematic reduction in negative interaction and communication was registered, but no attempt was done to separate the influence of a number of strategies aiming emotional vocabulary, communication, empathy and conflict resolution (Heydenberk and Heydenberk, 2007). In a recent longitudinal study of preschool vocabulary as a predictor of adolescent internalizing mental health problems, there were only weak and inconsistent predictions (Thornton et al., 2021). Despite this, vocabulary – specifically emotional vocabulary – has been shown to act as a resource for social function by improving prosocial behavior and reducing the risk for victimization and rejection in a six-month perspective (Miller et al., 2005).

The primary function of language is to communicate information of many kinds between people. Language is the use of words, signs or other symbolic representations organized in a structure (sentences and paragraphs) that carry information about logic and process between the elements. Word, phrases and sentences are used to express and learn about the world and ourselves. Words and sentence structure build on underlying cognitive structures; the conceptual organization of the content and dynamics of our understanding of the world and ourselves. Therefore concepts and knowledge are supposed to influence language, and language can be a tool for communication and reflection influencing our knowledge. These underlying cognitive structures are reflected in the elements and structures of language and must be partly shared in social environments as a prerequisite for meaningful communication, reflecting culture (Clauss, 1998).

In similar ways, emotional language can express emotions and inform us about the emotional reactions of others in social interplay, and attentive listening to our emotional speech can influence and change our emotions (Keaton et al., 2015). Thus, both positive and negative mental health (functioning and problems) could be related to language and communication, and possibly more to emotional language and communication. Therefore, a rich and nuanced emotional vocabulary may be beneficial for mental health by serving emotion regulation and social support, although there is also a possibility for using language in ways that create or aggravates mental health problems (Sala et al., 2014). Three hierarchical levels of emotional understanding have been identified: the external, mental and reflective level constituting a conceptual framework for emotional understanding (Pons and Harris, 2005). The external focus understanding of facial expressions, situational influences, and influence of events and reminders, the mental is about understanding the role of beliefs, desires and hiding/expressing emotions, and finally the reflective level focusing the understanding of multiple perspectives, mixed feelings regulation and rumination (Pons and Harris, 2005; Sprung et al., 2015). One of these external aspects of emotional understanding is emotional vocabulary; the knowledge and use of words and phrases describing emotional and affective states and from facial expressions. The development and training of similar skills have been shown to influence social competence and psychological and school adjustment (Denham et al., 2012; Metsala et al., 2017), and being impacted by non-verbal intelligence and learning difficulties, but not from abusive experience (Pons et al., 2014; Metsala et al., 2017; Barrett et al., 2019). Emotional vocabulary has been shown to be approximately doubled every second year through development, at least up to 12 years of age (Baron-Cohen et al., 2010), but with considerable individual differences in quantity which seem to remain stable through typical development as well as interventions and training (Pons and Harris, 2005; Pons et al., 2019).

Those receiving individual targeted mental health interventions – psychotherapy – may still change their position in the distribution of mental health as well as emotional understanding and communication skills – relative to those not receiving it. In psychotherapy; often called “the talking cure,” language has had a prominent role from the psychoanalytic use of free association through narrative and socio-constructive therapies to recent advances in cognitive-behavioral therapy and emotion regulation (Moloney, 2013), utilizing how emotional experience influence language and language shape interpretations of events and emotions. Often psychotherapy focuses on searching for word and narrative that can express and clarify experience, or shape and reinterpret them. In such processes poor emotional language can be an obstacle, and some treatment interventions can result in enrichment and development of vocabulary and communication skills (Moloney, 2013). In the psychotherapy literature the influence of alexithymia (Rottenberg and Gross, 2007; Preece et al., 2018) has addressed the observed associations between mental health problems and weak abilities to focus on, identify and describe emotions among adults (Nowakowski et al., 2013; Preece et al., 2018), especially for eating disorders, somatization and dissociation (Taylor et al., 1996), or more generally for any mental health problem involving emotion regulation (Ellard et al., 2010). Regrettably, alexithymia has not been investigated from a developmental perspective whereas, emotional vocabulary has been shown to be approximately doubled every second year through development, at least up to 12 years of age (Baron-Cohen et al., 2010).

In the present study we have therefore focused specifically on the associations between positive and negative mental health (specified as prosocial behavior and emotional, hyperactive or behavioral problems) and *emotional vocabulary*; specified as the quantity of words and phrases used to characterize facial emotional expressions in a free labeling task (Barrett et al., 2019). Emotional vocabulary is part of the external level of emotional understanding in Pons and Harris (2005) framework for emotional understanding (Pons and Harris, 2005), and thus only one of the candidates to explain the associations between language and mental health. The associations found for emotional vocabulary may well reflect general vocabulary or non-verbal intelligence, or communication skills utilizing several other more basic resources, but the literature does not always differentiate clearly between these aspects of cognitive, language and communication capacities when suggesting explanations for the empirical findings of associations between language and mental health.

There are relatively few studies investigating the specific role of emotional vocabulary in mental health (Fabes et al., 2001). Our hypothesis is that emotional vocabulary could be more important for mental health than general vocabulary, and reflect a specific resource beyond intelligence and communication skills. However, associations to emotional vocabulary may only reflect the importance of general vocabulary, non-verbal problem solving, age (maturation and development in general) or communication skills. Therefore, these other variables should be included to separate their importance from that of emotional vocabulary.

Emotional vocabulary has been shown to increase gradually in amount and differentiation with age through childhood and adolescence toward adulthood (Grosse et al., 2021), and facial expression processing are known to develop with age based on neurobiological maturation and socialization, with a female advantage (McClure, 2000). This trajectory is similar to the increase in general vocabulary, which is considered an aspect of general cognitive development, also increasing with age and showing gender differences (Doost et al., 1999). Therefore, when evaluating associations between emotional vocabulary and mental health, it is necessary to consider whether these reflect or interact with age, gender, cognitive abilities, general vocabulary or communication skills.

There is no gold standard for evaluating emotional vocabulary, and several of the existing assessments (Camras and Allison, 1985) focus on the ability to interpret emotional information, while others count the number of words used to describe imagined emotions as used by Doost (Doost et al., 1999). Other tests of emotional understanding focus on the understanding of emotions and emotional processes (Barrett et al., 2019), or the correct identification/recognition or use of emotional words. This can be done as choice-from-array tasks or free-labeling tasks. As we wanted to investigate the differentiation and richness of emotional vocabulary, we wanted to use a free-labeling tasks based on multiple drawings without any context or history attached. To test our hypothesis, we therefore developed a simple test of emotional vocabulary for use among school youths, counting the number of different emotion-related words elicited when presented to drawings of emotional faces. See the Methods section for elaboration of this.

Thus, the present study explores a small piece of the puzzle connecting language and mental health, comparing the associations between parent-reported child mental health symptoms and child emotional vocabulary, compared to age, general vocabulary, non-verbal problem solving and parent-reported social communication skills. Finally, the associations between emotional vocabulary and these other variables will be explored, expecting substantial positive associations between them.

## Materials and Methods

### Sample

At two schools serving their entire local population in a rural county without any private or special schools, 410 students and their parents were invited to participate. No inclusion or exclusion criteria were used, so the sample represents the full population variation of all students because any disorders or disabilities were included. Of these, the parents of 71 (17%) youths aged 10–16 agreed to participate, and 47% of the students were girls. The area was characterized by farms, small villages and small businesses in various trades and some academic employment, with a socio-economic level slightly below national average. The number of participants were not dictated by a power analysis, but rather by the practical and resource contraints following from using two masters students to conduct the data-collection.

### Instruments

The participants’ communicative competence was assessed using the Norwegian translation of the Children’s Communication Checklist (CCC-2) (Helland et al., 2009), an instrument that was developed to assess the pragmatic aspects of communication problems among children based on parent or teacher reports (Bishop, 1998). The first version (CCC) was connected to normative community samples in England (Bishop and Baird, 2001), and a revised version was published as CCC-2 (Children’s Communicative Checklist, 2.ed.) (Bishop, 2003). It consists of 10 subscales (7 items pr. subscale): (A) speech, (B) syntax, (C) semantics, (D) coherence, (E) inappropriate initiation, (F) stereotyped language, (G) use of context, (H) non-verbal communication, (I) social relations, and (J) interests. The ‘Global Communication Composite’ (GCC) sum score based on the A-H subscales indicating communication skills, the Pragmatic Composite D-H subscale indicating pragmatic language impairment or *pragmatic language skills*, whereas the Social Interaction Deviances Composite (A + B + C + D)-(E + H + I + J) has been used as a measure of specific language impairment (Norbury et al., 2004; Bishop et al., 2006).

Child psychiatric samples from Britain and Norway showed mean scores that were approximately two standard deviations lower than community sample means in both countries (Bishop and Baird, 2001; Helland and Heimann, 2007; Brenne and Rimehaug, 2019). The internal consistency of the Norwegian CCC-2 is reported as good [Cronbach’s alpha ranging from 0.73 to 0.89 for the subscales; (Helland et al., 2009)]. Pragmatic skills develop through childhood, but the GCC index does not show strong associations with age in middle childhood and does not show gender differences (Helland et al., 2009; Brenne and Rimehaug, 2019). Age and gender norms are not established.

General vocabulary was assessed using the vocabulary test from Wechsler Intelligence Scales for Children, 4th edition (WISC-IV Vocabulary). WISC-IV Vocabulary contributes to the verbal and total intelligence scores. Normative scores are available and show a strong increase with age, and girls tend to score higher than boys (Wechsler, 2003). Age-standardized vocabulary (scaled scores in WISC-IV) can be used as a simple indicator of verbal intelligence. Both raw scores (*general vocabulary*) or standardized scores (*verbal intelligence*) will be used in the analysis depending on other variables and the purpose of the analysis.

Non-verbal problem solving was measured with Raven’s Standard Progressive matrices. The scores of the Raven are known to increase strongly with age, and percentile scores depending on age are available as a method of standardization (Raven et al., 1988). Non-verbal problem-solving percentiles can be used as a simple indicator of non-verbal intelligence. Raw scores (*non-verbal problem solving*) or age-relative percentiles (*non-verbal intelligence*) will be used depending on the purpose of the analysis.

Mental health was measured using the one-page parent version of the Strengths and Difficulties Questionnaire (SDQ; Goodman, 2009). The SDQ is a brief measure used to assess mental health, including an assessment of prosocial behavior in children. Its 25 items are scored on a 3-point Likert scale and contribute to five subscales (Emotional Problems, Conduct Problems, Hyperactivity-Inattention, Peer Problems, summed into the Total Difficulties score used as a measure of *mental health problems*, and the Prosocial Behavior scale is considered an aspect of *positive mental health*. SDQ problem scores do not show any strong association with age, although association with age has been reported in some studies (Van Roy et al., 2006). Previous validations have demonstrated satisfactory reliability (internal consistency and test-retest reliability) and validity (Stone et al., 2015), and in the present study, internal consistency was found to be acceptable.

Emotional Vocabulary was tested by presenting 32 drawings of facial emotional expressions by Jim Borgman (2017) (see Supplementary Materials) one at a time asking each child to describe what the child in the drawing was feeling – a free-labeling method (Barrett et al., 2019), but without evaluating correctness. This was done to reflect the richness and differentiation of emotional vocabulary rather than correct labeling. Therefore the number of different emotional words and phrases used during the entire test was counted. Thus, if the same word was used connected to several drawings, this did not increase the word count. Multiple words and phrases were allowed and counted for each facial drawing to be used in split-half reliability calculations. Words and phrases connected to affective states like “exhausted,” “excited” and “sick” representing different levels of valence and arousal were counted in addition to those connected to the classical emotional categories ‘happiness, “sadness,” “fear.” “anger,” “surprise,” and “disgust” (Barrett et al., 2019). Slang or ideosyncratic words were accepted, but not words describing a related behavior or mental activity without emotional valence, additions of an amplifying or specifying prefix, or a negation of already used words. No attempt was made to group the responses in content categories. Psychometric properties and associations with age and gender are reported in the Results section rather than Methods because this is an instrument under development. Others have shown an increase in number and differentiation in emotional vocabulary with age (Doost et al., 1999). Since no proper age norms for emotional vocabulary were available to be compared to or to be used in age-standardized conversions of this variable was not possible.

### Procedure

Youths and their parents were invited to the study by their contact-teacher, providing that this teacher had volunteered to assist with the recruitment and data-collection. Parents consenting to participate were supplied with CCC-2 and SDQ paper-and-pencil questionnaires distributed by mail by the researchers and returned by mail to a local university. The other instruments were individually administered to youths at school during school hours by university students experienced in work with youths. These were the test from WISC-IV; Vocabulary, Raven’s Standard Progressive Matrices. The emotional vocabulary test was conducted in the classroom, where each student recorded their individual response on paper while the drawings were presented slowly one-by-one on a large screen.

### Statistics

The SPSS statistical program, version 24, was used to analyse the data, calculate descriptive data, and conduct bivariate correlations, one-way ANOVA, reliability indices, and linear regression.

The correlations involving general vocabulary or non-verbal abilities were primarily calculated for these raw scores of abilities, and if standardized scores relative to age (verbal and non-verbal intelligence) were used, this is specified.

### Ethics

The youths and their parents were recruited through their contact teacher at school depending on parental consent. Both youths and parents were informed and contributed as informants. Each school was rewarded with 10 US$ per participating child, earmarked as means for improving joint social activity at school. Youth and parent were not individually compensated. The study was approved by the Regional Medical Research Ethics Committee (REK approval 26631).

## Results

### Emotional Vocabulary

When the emotional drawings were split in half randomly or as every second drawing assigned to each half, the emotional vocabulary word counts showed split-half Cronbach’s alpha of 0.66 and 0.79, which is acceptable. The emotional vocabulary counts ranged from 10 to 30 (see descriptive overview of variables in Table 1, and description of age groups on emotional vocabulary in Table 2).

**TABLE 1:** Descriptive statistics.

	N	Minimum	Maximum	Mean	S.D.
Age	71	10 year 5 month	15 y 3 mth	12.8	1.3
Vocabulary	71	10	42	24.3	6.7
Verbal intelligence	71	1	11	5.6	2.3
Non-verbal problem solving (Sums)	71	20	59	46.5	6.4
Non-verbal intelligence (Percentiles)	71	1	99	69.1	24.3
Emotional vocabulary	70	10	30	19.2	5.1
Communication composite (GCC A-H)	52	23	96	76.4	16.0
Pragmatic composite (D-H)	52	13	61	47.7	11.0
Social interaction deviance composite	52	–8	13	–0.23	4.5
Language skills (ABCD)	53	12	48	38.5	8.5
Social skills (EHIJ)	52	8	50	38.8	9.6
SDQ emotional problems	56	0	9	2.3	2.2
SDQ behavioral problems	56	0	4	1.1	1.2
SDQ hyperactive problem	56	0	7	2.8	2.0
SDQ social problems	56	0	7	1.0	1.4
SDQ total problems	56	0	21	7.2	5.0
SDQ prosocial scale	56	4	10	8.4	1.4

**TABLE 2:** Emotional vocabulary count distribution in age-groups.

Age	11	12	13	14	15
N	10	16	15	23	6
Mean	16.2	16.7	19.2	22.0	19.7
s.d.	4.7	4.3	3.9	5.0	5,7
Range	11–23	10–24	13–28	13–30	14–30

### Associations With Age and Gender

Gender was only significantly associated with two of the study variables insofar as girls scored significantly higher on emotional vocabulary [*F*(1,68) = 7.930, *p* = 0.006, eta^2^ = 0.104)] and emotional problems [*F*(1,54) = 10.205, *p* = 0.002, eta^2^ = 0.159]. As expected, age showed significant linear associations with general vocabulary (*r* = 0.35, *p* < 0.005), emotional vocabulary (*r* = 0.38, *p* < 0.001) and non-verbal problem solving (*r* = 0.39, *p* < 0.001), but there were no significant associations between age and pragmatic language or mental health symptoms.

### Associations Between Study Variables

As expected, emotional vocabulary was significantly associated with general vocabulary (*r* = 0.42, *p* < 0.005) and non-verbal problem solving (*r* = 0.32, *p* < 0.01). Non-verbal problem solving and general vocabulary were also significantly associated (*r* = 0.37, *p* < 0.005). In contrast, pragmatic skills were not significantly associated with any of the three abilities: general or emotional vocabulary or non-verbal problem solving.

### Associations to Mental Health Problems

Table 3 shows the correlations between the four abilities and the mental health scales. Only pragmatic skills show some significant associations with mental health: negative associations with the problem total score, and hyperactivity and behavioral problems scales, and a positive association with the prosocial scale. Emotional vocabulary was only significantly associated with emotional problems, but in the opposite direction of what we expected (*r* = 0.30, *p* < 0.05), which means that there was an association between larger emotional vocabulary and more emotional symptoms reported by parents. Non-verbal problem solving and general vocabulary were not significantly associated with any mental health aspects.

**TABLE 3:** Strength and difficulties questionnaire (SDQ): Mental health aspects; correlations to abilities.

	SDQ emotional	SDQ behavioral	SDQ hyperactivity	SDQ social	SDQ total	SDQ prosocial
Emotional vocabulary	0.295^*^	0.170	0.005	0.006	0.177	0.046
General vocabulary	0.004	0.185	–0.051	0.139	0.064	0.105
Verbal intelligence^#^	–0.112	0.124	–0.155	–0.118	–0.114	0.280*
Non-verbal problem solving	0.026	–0.144	–0.086	0.222	0.002	–0.049
Non-verbal intelligence^#^	–0.017	–0.229	–0.166	0.002	–0.103	0.038
Communication composite	–0.175	–0.537^***^	–0.355*	–0.208	–0.406^**^	0.387^**^

Bold type figures, Statistically significant results; * p < 0.05, ** p < 0.005, *** p < 0.001. ^#^ Skills relative to age standards.

The age-standardized variables (verbal intelligence and non-verbal intelligence) were not significantly associated with any mental health problems, whereas verbal intelligence was positively associated with prosocial skills (see Table 3).

### Combined Regression Models

The mental health scales were regressed on a combined model including age, gender, non-verbal problem solving, and general and emotional vocabulary to explore their partial associations and combined influence on mental health. This regression (summarized in Table 4) resulted in a similar picture as the correlation analyses. In short, the main influence was that pragmatic language significantly predicted some aspects of mental health, but not all. The other abilities did not show a significant influence on mental health. A blockwise reanalysis showed that the change in model predictive value did not increase significantly for any mental health outcome by adding vocabulary, emotional vocabulary and non-verbal problem solving as a block to the regression model with age, gender and pragmatic language (see Table 5).

**TABLE 4:** Model 1: mental health scales regressed on age, gender, school, vocabulary, emotional vocabulary, non-verbal problem solving, and communication skills. Standardized beta with *p*-value level and model statistics.

Standardized beta	Emotional problems	Behavioral problems	Hyperactive symptoms	Social problems	Total problems	Prosocial scale
Age	0.269	0.058	0.333^*^	0.299	0.350^*^	–0.295
Gender	–0.417^**^	–0.217	–0.330^*^	–0.118	–0.404^**^	–0.050
School	0.131	0.193	0.020	–0.173	0.069	0.024
Communication composite	–0.142	–0.492^***^	–0.364^*^	–0.239	–0.392^**^	0.358^*^
Emotional vocabulary	0.070	–0.069	–0.200	–0.165	–0.111	0.093
General vocabulary	–0.141	0.213	0.072	0.061	–0.023	0.202
Non-verbal problem solving	–0.014	–0.170	–0.142	0.054	–0.089	–0.008
Adjusted R-squared	0.180	0.339	0.152	0.056	0.261	0.123
Regression *F*(df = 7/42)	2.528	4.596	2.254	1.413	3.471	1.986
Regression significance	0.028	0.001	0.048	0.226	0.005	0.080

Bold type figures, Statistically significant results; * = p < 0.05, ** p < 0.005, *** p < 0.001.

**TABLE 5:** Prediction of mental health by Model 1: pragmatic language. age and gender and change in prediction when adding general vocabulary emotional vocabulary and non-verbal intelligence into Model 2. Model and change statistics.

Adjusted R^2^	Emotional Problems	Behavioral Problems	Hyperactive Symptoms	Social Problems	Total Problems	Prosocial Scale
Model 1 gender age school & Communication	0.217^**^	0.335^***^	0.133^*^	0.102	0.288^***^	0.129*
Model 2 (Model1 + Vocabulary, Emotional vocabulary and Non-verbal problemsolving)	0.180^*^	0.339^***^	0.153^*^	0.056	0.261^**^	**0.123**
Model change adjusted R2	–0.037	0.004	0.019	–0.046	–0.027	–0.006
Model change *p*-value	0.813	0.363	0.278	0.848	0.713	0.445

Bold type figures, Statistically significant results; * = p < 0.05, ** p < 0.005, *** p < 0.001.

The participating youths from one of the two schools showed significantly higher non-verbal intelligence, fewer behavioral problems, larger emotional vocabularies and consisted of more boys. Introducing “school” into the regression models did not significantly influence any of the analyses and did not alter any conclusions. Therefore these supplementary analyses as not reported in detail.

Finally, the correlations between mental health subscales and the communication subscales were explored (see Table 6). The main pattern is that all correlations to subscales, significant or not, have consistent directions, and that the correlations between mental health and composite scales are parallelled without any clear differences, except a lack of associations to the social interaction deviance composite (SIDC). The only subscale significantly correlated to social problems was Coherence.

**TABLE 6:** Correlations between aspects of mental health – the Strengths and Difficulties questionnaire (SDQ), subscales and composite scores of the Children’s Communication Checklist 2 (CCC-2).

	Emotional problems	Behavioral problems	Hyperactive problems	Social problems	SDQ total problems	Prosocial behavior
CCC-2 A Speech	–0.189	–0.563^***^	–0.363^**^	–0.270	–0.440^***^	0.319^**^
CCC-2 B Syntax	–0.096	–0.379^**^	–0.162	–0.078	–0.178	0.547^***^
CCC-2 C Semantics	–0.067	–0.312^*^	–0.113	–0.053	–0.167	0.020
CCC-2 D Coherence	–0.085	–0.347^*^	–0.282^*^	–0.304^*^	–0.319^*^	0.232
CCC-2 E Inappropriate initiation	–0.166	–0.431^**^	–0.291^*^	–0.197	–0.348^*^	0.257
CCC-2 F Stereotyped conv.	–0.250	–0.398^**^	–0.325^*^	–0.120	–0.367^*^	0.285^*^
CCC-2 G Use of context	–0.111	–0.403^**^	–0.442^***^	–0.055	–0.337^*^	0.431^**^
CCC-2 H Rapport	–0.164	–0.430^**^	–0.225	–0.161	–0.311^*^	0.380^**^
CCC-2 I Social	-0.046	–0.417^**^	–0.255	–0.139	–0.264	0.434^***^
CCC-2 J Interests	–0.247	–0.436^***^	–0.260	–0.122	–0.351^*^	0.292^*^
**CCC-2 Composite scales**						
General communication (A-H)	–0.175	–0.537^***^	–0.355^**^	–0.208	–0.406^**^	0.387^**^
Pragmatics (D to H)	–0.177	–0.492^***^	–0.374^**^	–0.232	–0.410^**^	0.388^**^
Social interaction deviance	0.135	0.155	0.074	0.035	0.135	–0.309^*^
Language skills (A-D)	0.138	–0.510^***^	–302*	–0.208	–0.364^*^	0.315^*^
Social skills (EHIJ)	–0.184	–0.533^***^	–0.303*	–0.210	–0.390^**^	0.432^***^

Bold type figures, Statistically significant results; * = p < 0.05, ** p < 0.005, *** p < 0.001.

## Discussion

The results indicate that emotional vocabulary in school youths is not a general resource for mental health, and the same conclusion applies to general vocabulary and non-verbal problem solving (or the age-standardized scores; verbal/non-verbal intelligence) – except for a weak significant positive association between verbal intelligence and prosocial behavior. However, pragmatic language – the skills to communicate effectively – was negatively associated with the behavioral and hyperactivity problems, but not with emotional and social problems. Pragmatic skills were also positively associated with prosocial behavior, indicating that pragmatic skills are a resource factor for positive aspects of mental health. Emotional vocabulary was positively associated with parental reports of emotional problems – in contrast to our hypothesized negative association. The regression models produced similar conclusions as indicated by the correlation matrix, suggesting that none of the associations reflected confounding influence.

Emotional vocabulary as measured by counting the number of emotional words and phrases elicited by 30 facial drawings, correlated moderately with general vocabulary and showed good split-half reliability. Furthermore, emotional vocabulary correlated with age and non-verbal problem solving but did not show significant gender differences. This innovative measure therefore behaved as expected, and split-half reliability with criterion validity.

In sum, emotional vocabulary did not show a stronger association to mental health than general vocabulary, and no significantly additional value to that indicated by the associations between communication skills and mental health. Due to the limited sample size, this could be a false negative conclusion, however, even the non-significant association tendencies do not point in a consistent direction. The predictive value of communications skills is not significantly or consistently improved by adding all the other skills (emotional vocabulary, general vocabulary and non-verbal problem solving) to the predictive model, indicating that these skills only have marginal significance for mental health, if not resulting in improved communication skills.

The ten subscales of CCC-2 and the five composite scores included in our analyses did not indicate any clear specificity between aspects of CCC-2, although there were some differences between CCC-2 subscales in their associations to mental health. We find it difficult to suggest any interpretation of these differences beyond that most associations are stronger that those found for vocabulary and emotional vocabulary, and than the consistent directions suggest that many aspects of communication contributes to the associations to mental health. Only one tendency was very clear: Emotional problems were not associated in the expected negative direction to any aspect of communication or vocabulary (emotional or general). Only one significant positive correlation was found between emotional vocabulary and parent-reported emotional problems, suggesting that youths with a rich emotional vocabulary communicate their emotional problems more to others, so that parents become aware of them more easily and are able to report them.

The main finding that communication skills; the active use of vocabulary in communication are negatively associated with externalizing problems and hyperactive problems, suggest that communication skills can partly protect against externalizing problems by enabling verbal expressions of presumably underlying difficulties, and thereby be heard and be able to influence their surroundings without resorting to acting out behavior. The expectation that emotional vocabulary could be more important than vocabulary in general for mental health was not supported because neither of them showed significant negative associations to mental health. Even the combined influence of non-verbal problem solving, general and emotional vocabulary did not increase the ability to predict externalizing mental health problems beyond the prediction from age, gender and communication skills.

Despite these lacking general associations between emotional vocabulary and mental health, it is still possible that emotional vocabulary has specific importance for some problem types, such as eating disorders, and dissociative and somatization disorders (Taylor et al., 1996) and not so much for the limited selection of more ordinary problems included in the SDQ. It is also possible that vocabulary resources can influence mental health mediated through communications skills, but the present cross-sectional data were not suitable for analyzing mediation or moderation models for development of, or recovery from, mental health problems. Future research should consider whether language and vocabulary only have indirect and small relevance for mental health, and that an ordinary socially functioning language is sufficient, with no additional gain for mental health from an enriched and nuanced emotional language – “more is not necessarily better”. One person’s enriched language may profit internal mental processes, but would not necessarily benefit communication with others with only typical language capacity.

Among the minor findings, vocabulary and non-verbal problem solving was also converted to age-relative scores as simple indicators of verbal and non-verbal intelligence did not make any difference for the results. These conversions could be interpreted as general developmental level or at least cognitive developmental level. Interestingly, neither of these conversions showed significant associations with any aspect of mental health included in this study, or even a tendency toward stronger associations than the raw scores.

It was expected that a richer emotional vocabulary could protect against or enable youths to cope with emotional problems; however, emotional vocabulary only showed an unexpected positive association with emotional problems, as reported by parents. These results can be interpreted as indicating that a rich emotional vocabulary enables youths to convey their problems to their parents so that parents know about and are able to report them, rather than contribute to a lower symptom level. Another possible interpretation is that emotional problems that have been known and acknowledged by parents have also been discussed repeatedly and resulted in a richer emotionally vocabulary among children with emotional problems.

Measuring the quantity of emotional vocabulary seems possible by counting the spontaneous words elicited to drawings of emotional facial expressions. This simple approach is reliable, as indicated by the split-half statistics. Its validity is confirmed by the association with general vocabulary, although the vocabulary test probes a correct understanding of ordinary words rather than simply knowing them. Our test of emotional vocabulary is a possible method for evaluating and producing age norms for youth’s emotional vocabulary size at different ages in larger samples (Preece et al., 2018). However, there is also a need to develop more elaborate scoring rules for what can be counted as separate emotional words.

### Clinical Implications

Systematic preventive efforts to strengthen emotional vocabulary may help language development and could indirectly improve communication skills by increasing the degree of differentiation between emotional states cognitively as well as in communication. However, a positive influence on mental health from the quantity of emotional vocabulary was not confirmed, and no associations were found between mental health, general vocabulary and non-verbal problem solving. This suggests that simple expansion of emotional or general vocabulary will probably not improve general mental health without integrating training and improving social communication skills.

Despite this negative finding, emotional vocabulary may still deserve the development of a structured instrument and empirical age norms for quantifying emotional vocabulary as a supplement to a correct understanding of emotional words and concepts.

### Strengths and Limitations

The major strength of this study is that several aspects of development and communication were evaluated simultaneously so that their separate and combined relevance for mental health could be evaluated.

It was an important weakness that it is a weakness that mental health is only reported by parents without supplementing self-reports. Comparing self-reports to parent-reports could have aided the interpretations, and regarding internalized/emotional problems self-reports are considered more valid than observation-based information from others. It is also a weakness that the SDQ represents a rather limited selection of mental health problems, although some major aspects are represented. Furthermore, it was a weakness that the sample was not large and representative enough to be used for preliminary norms for emotional vocabulary and the study did not follow the developmental or measurement stability of emotional vocabulary over time longitudinally. However, testing associations does not require highly representative samples. The sample size was somewhat small, so there is a danger of drawing false negative conclusions due to low statistical power. Finally, it was a weakness that no other aspects of emotional understanding than emotional vocabulary were investigated.

## Conclusion

Emotional vocabulary was not significantly associated with parent-reported mental health. Further research should investigate whether this has implications for interventions specifically teaching or training emotional understanding. The suggestion is that expanding emotional vocabulary *per se* is unlikely to improve mental health unless vocabulary expansion is integrated with improvements in social communication as a practical social skill.

## Data Availability Statement

The raw data supporting the conclusions of this article will be made available by the authors, without undue reservation.

## Ethics Statement

The studies involving human participants were reviewed and approved by Regional Medical Research Ethics Committee (REK approval 26631). Written informed consent to participate in this study was provided by the participants’ legal guardian/next of kin.

## Author Contributions

TR conceived the study, conducted the analyses, and drafted the manuscript. Both authors contributed significantly to the article and approved the submitted version.

## Conflict of Interest

The authors declare that the research was conducted in the absence of any commercial or financial relationships that could be construed as a potential conflict of interest.

## Publisher’s Note

All claims expressed in this article are solely those of the authors and do not necessarily represent those of their affiliated organizations, or those of the publisher, the editors and the reviewers. Any product that may be evaluated in this article, or claim that may be made by its manufacturer, is not guaranteed or endorsed by the publisher.
