# Supplementary material for: Communication and Emotional Vocabulary; Relevance for Mental Health Among School-Age Youths
Source: Front Psychol. 2022 Apr 25;13:847412. doi: 10.3389/fpsyg.2022.847412 (PMC9083540; doi:10.3389/fpsyg.2022.847412)
Supplement: Supplementary file 1 [file Data_Sheet_1.PDF]

EMwords © Drawings: Creative Therapy Associates
Prepared 2016 by T. Rimehaug, NTNU, Norway

INSTRUCTIONS FOR GROUP ADMINISTRATION

Preparations: If 1-2 students does not have sufficient writing skills to record their answers themselves, an adult may assist them by writing down their vocal answers

Introduction:
BEFORE any drawings are shown:
"I am going to show you drawings of a boy.
I want you to think about wht is happening inside him. What does his facial expression mean."

Facial drawings are show none by one on a large TV / video-projector **20 seconds**
For the 1. and 2. drawing: Show the drawing first and then say:

"How do you think the boy is feeling, how is he inside, what does his facial expression mean.
Write down the words or expression you think of by the drawing number.
There are noe correct or wrong answers"

When drawing 3-4 are shown, say:

"Write down the words and sentences you think of.
If you do not find anythink to write down, only write an X

Repeat this last instruction after drawing nr. 10, 15, 20, and 25.

After all drawings have been shown:

«Thank you for your efforts. You are now finished»

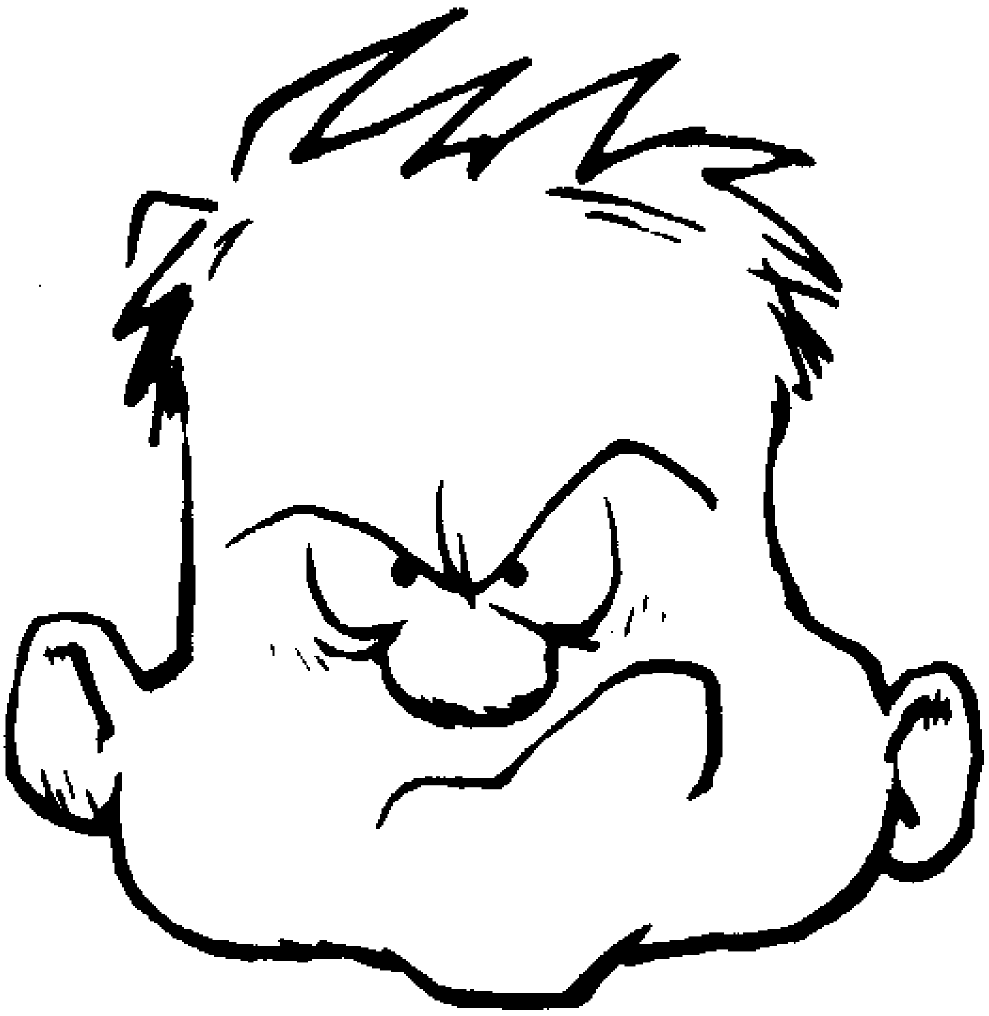

13

(Example drawing from Jim Borgman – 29 more on
http://www.ctherapy.com/feelings/feelings-posters/

© Drawings: Creative Therapy Associates)

SCORING INSTRUCTION:

Count

- The number of different words or expressions (short sentences) elicited by each child describing emotions or affective states.
- Synonyms count, but not «bending» or adding prefixes or adjectives regulating degree.
- Affective states like energy-level and discomfort that are general though body-related.
- Slang or ideosyncratic words/expressions.
- Multiple words and different content to one drawing

Do NOT count

- Repetitions of identical words/expressions to later drawings.
- Behaviors and expressions alone without emotional or affective aspect do not count (grimace or blushing does not count, funny face or embarrassed count, terribly angry does not count in addition to angry).
- Longer sentences without a clear emotional/affective content does not count.

THE ARTISTS' WORDs CONNECTED TO EACH DRAWING; THE ORIGINAL INTENTION, BUT NOT A NORM FOR RIGHT/WRONG ANSWER

	English	Norwegian
1	Shy	Forsiktig
2	Embarrassed	Brydd
3	Mischievous	Ondskapsfull
4	Cautious	Vaktsom
5	Disgusted	Kvalm
6	Smug	Innbilsk
7	Sad	Trist
8	Hysterical	Hysterisk
9	Anxious	Engstelig
10	Bored	Kjeder seg
11	Confused	Forvirret
12	Lovestruck	Forelsket
13	Angry	Sint
14	hopeful	Håpefull
15	Guilty	Skyldig

16	Exhausted	Utmattet
17	Suspicious	Mistenksom
18	Lonely	Ensom
19	Overwhelmed	Overveldet
20	Jealous	Sjalu
21	Ecstatic	Ekstatisk
22	Surprised	Overrasket
23	Shocked	Sjokkert
24	Frustrated	Frustrert
25	Confident	Selvtilfreds
26	Depressed	Deprimert
27	Frightened	Skremt
28	Enraged	Rasende
29	Ashamed	Skamfull
30	Happy	Glad
